# Template-Free Wet-Spinning of Multifunctional Sodium Alginate Hollow Hydrogels

**DOI:** 10.3390/gels12030224

**Published:** 2026-03-10

**Authors:** Na Pan, Haoran Sun, Yanhu Zhan

**Affiliations:** 1 School of Materials Science and Engineering, Liaocheng University, Liaocheng 252000, China; zhanyanhu@lcu.edu.cn; 2 School of Chemistry and Chemical Engineering, Liaocheng University, Liaocheng 252000, China; qwerty451018@163.com

**Keywords:** sodium alginate, hollow hydrogels, wet-spinning, template-free fabrication, ionic cross-linking, CO_2_-induced hollowing architectures

## Abstract

Hollow hydrogels are promising for flexible electronics and bioengineering, yet their fabrication is limited by sacrificial templates, specialized equipment, and complex engineering processes. Herein, a facile wet-spinning strategy is developed to fabricate sodium alginate (SA) hollow hydrogels. Extruding SA/CaCO_3_ precursor suspension into an acidic coagulation bath induces simultaneous ionic cross-linking and in situ CO_2_ generation, driving the self-formation of hollow tubular architectures with tunable morphologies, mechanical performance, macroscopic architecture, and functional properties. Moreover, the introduction of secondary cross-linking enhances the SA hydrogels’ water retention and resistance to freezing conditions. Utilizing their intrinsic ionic conductivity, the hollow hydrogels demonstrate outstanding sensing performance, enabling reliable detection of both large-amplitude limb motions and subtle muscle activity in the human body. Furthermore, hollow hydrogel tubes with diverse geometries can be readily fabricated by simply modifying the spinning mold, thereby broadening their potential applications. In vitro cytotoxicity assessments further confirm that the SA hollow hydrogels exhibit excellent biocompatibility with minimal cytotoxicity, satisfying the fundamental criteria for bioengineering applications. The combination of a simple yet controllable fabrication strategy with the intrinsic multifunctionality of the SA hollow tubes confers substantial potential for their deployment in bioengineering and flexible electronic applications.

## 1. Introduction

Hydrogels are three-dimensional, hydrophilic polymeric networks characterized by high water content, pronounced hygroscopicity, mechanical compliance, and exceptional extracellular matrix (ECM)-mimicking characteristics [1,2,3]. These attributes have positioned hydrogels as highly attractive materials for a wide range of applications, including soft actuators, robotic systems, flexible electronic devices such as strain sensors, and wearable platforms, as well as biomedical technologies, notably drug delivery and tissue engineering [4,5,6,7]. The architectural configuration and geometric design of hydrogels play a critical role in governing their physicochemical properties and functional performance. In particular, hollow tubular hydrogels have attracted growing interest owing to their vessel-like architectures, inherent permeability, geometrical tunability, and programmable functionality, rendering them highly attractive for advanced applications [8,9,10,11]. However, fabricating hollow hydrogels with well-defined architectures and integrated multifunctionality remains challenging, thereby constraining their practical implementation and large-scale application.

At present, extensive efforts have been devoted to the fabrication of hollow hydrogel structures. Among the available approaches, co-axial microfluidic spinning has emerged as a simple and versatile strategy for producing hydrogel microtubes, encompassing alginate-based and composite systems as well as gelatin methacryloyl (GelMA)-based hydrogel microtubular architectures [9,12,13,14,15,16,17,18]. Despite these advances, the resulting hydrogel microtubes generally exhibit insufficient mechanical robustness, which severely restricts their long-term stability and performance in biological environments. Alternatively, direct extrusion of highly concentrated prepolymer solutions through co-axial nozzles can produce high-strength hydrogel tubes. However, this strategy is inherently limited in its ability to produce complex, branched, hollow architectures. In contrast, three-dimensional (3D) printing has emerged as an effective technique for precisely fabricating uniform hollow hydrogel structures with controlled geometries [19,20,21]. However, the stringent demands for sophisticated instrumentation, complex printing protocols, and tightly controlled material formulations frequently impede the facile, scalable production of hollow hydrogel structures [22]. Template-assisted fabrication strategies, such as layer-by-layer assembly, electrodeposition, photopolymerization, thermal polymerization, and micromolding, have also been employed to construct hydrogel tubular structures [23,24,25,26,27,28]. However, the subsequent removal of sacrificial templates often induces structural damage, thereby weakening the integrity and reliability of the resulting hollow hydrogels [26,27,28].

Despite their widespread adoption, these fabrication strategies continue to encounter fundamental scientific and technical limitations, including: (i) achieving precise control over tubular dimensions and geometries; (ii) imparting high mechanical strength while maintaining adequate elasticity; (iii) enabling the straightforward construction of complex 3D hydrogel tubular ensembles; and (iv) realizing the integration of multiple functional attributes within a single structure. Accordingly, there is a strong need for a simple, scalable fabrication strategy for multifunctional hollow hydrogels, which requires careful selection of suitable precursor materials and an innovative processing approach.

Sodium alginate (SA), a naturally occurring anionic polysaccharide extracted from brown algae, has emerged as a highly attractive hydrogel precursor owing to its excellent biocompatibility and biodegradability, thereby avoiding the cytotoxicity concerns commonly associated with synthetic polymer systems [29,30]. The rapid ionic cross-linking of SA (e.g., via Ca^2+^ coordination) facilitates in situ gelation. At the same time, its abundant carboxyl functionalities impart high ionic conductivity, collectively providing a robust platform for multifunctional integration [31,32]. In addition, SA-based hydrogels offer readily tunable mechanical properties and cost-effectiveness, further reinforcing their suitability for practical applications across diverse fields.

In previous research, Zhang et al. fabricated SA tubes via a template-free film-to-tube conversion route in a CuSO_4_/H_2_O_2_/Tris–HCl system. While effective, this method inevitably introduces cytotoxic Cu^2+^ ions and relies on a two-step film-to-tube transformation process [33,34]. To address these issues and fully exploit the intrinsic properties of SA, we herein develop a simple, one-step, and template-free approach for constructing hollow SA tubes. This fundamental innovation in chemical implementation not only completely avoids the use of heavy metal ions but also eliminates the need for the pre-fabrication of films, significantly streamlining the fabrication process while ensuring biocompatibility. Specifically, our method involves immersing the spun precursor mixture into an external acid medium, where coupled external ionic cross-linking and internal gas evolution synergistically drive the formation of hollow tubular structures. The morphology and wall thickness of the hollow tubes can be readily modulated by tuning the solution pH and CaCO_3_ content, while their mechanical performance can be systematically adjusted to satisfy the demands of specific application scenarios. Furthermore, the hollow hydrogels display multiple integrated functionalities (including anti-drying, anti-freezing, and sensing capabilities), and enable the construction of increasingly complex hollow architectures, such as tortuous single-channel, bifurcating, and quadrifurcate vasculature-like networks. Together, this combination of methodological innovation and multifunctional integration emphasizes their considerable potential in flexible electronics and advanced biomedical engineering.

## 2. Results and Discussion

### 2.1. Fabrication and Characterization of the SA Hollow Hydrogel

**Figure 1:**
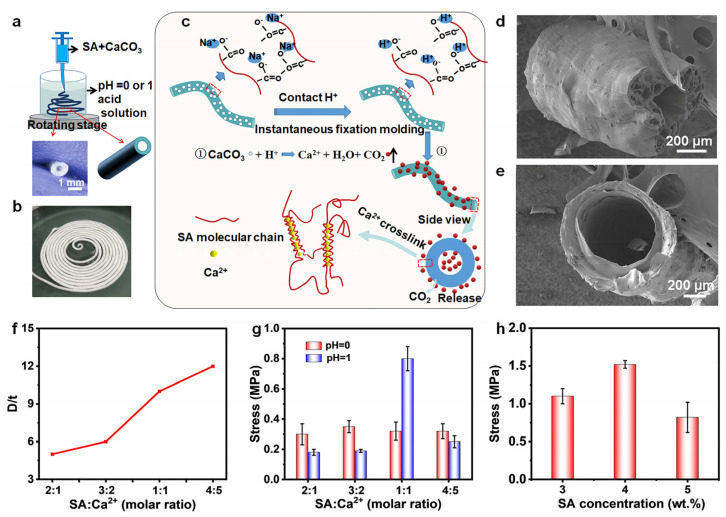
(**a**) Schematic illustration of the SA hollow hydrogel preparation process. (**b**) Mass production of SA hollow hydrogels. (**c**) Schematic illustration of the formation mechanism of SA hollow hydrogel. (**d**) SEM image of hollow hydrogel at pH = 0 (theoretical, 1 mol/L HCl). (**e**) SEM image of hollow hydrogel at pH = 1 (theoretical, 0.1 mol/L HCl). (**f**) Effect of the molar ratio of SA to calcium ions on the diameter and wall thickness ratio (D/t) of hollow hydrogels (pH = 1). (**g**) Effect of the molar ratio of SA to calcium ions on the mechanical strength of hollow hydrogels. (**h**) Effect of SA concentration on the mechanical strength of hollow hydrogels (pH = 1, SA:Ca^2+^ = 1:1).

As schematically depicted in Figure 1a, SA hollow hydrogels were fabricated by extruding an SA/CaCO_3_ spinning mixture into hydrochloric acid coagulation baths at theoretical pH 0 and 1. Following extrusion, the samples were maintained in the acidic gelation medium, allowing their transformation into interconnected hollow tubular structures, as confirmed by the corresponding optical images. Consequently, this approach is readily scalable and well suited for the large-scale production of hollow SA hydrogels (Figure 1b). Notably, the underlying formation mechanism of the hollow structures differs fundamentally from those of conventional 3D printing and co-axial microfluidic techniques. In the present strategy, hollow architecture generation proceeds through two distinct yet coupled processes, as schematically illustrated in Figure 1c. Upon contact between the spinning mixture and the acidic coagulation bath, rapid interfacial cross-linking occurs through protonation of the carboxyl groups of SA by H^+^ ions, effectively suppressing alginate dissolution in the aqueous medium. Notably, the pH values of the acidic solutions employed are lower than the pKa values of the SA constituent monomers, namely (β-D)-mannuronic acid (pK_a_ ≈ 3.38) and (α-L)-guluronic acid (pK_a_ ≈ 3.65), thereby ensuring efficient proton-mediated gelation [35,36]. In the strong acids (HCl), rigid shapes are obtained at pH values below 2. At higher pH values (2, 3, 4, 5), insufficient protons are available to liberate calcium ions, inhibiting the cross-linking of the hydrogel (Figure S1). Under these conditions, the carboxylate groups along the SA backbone undergo protonation, leading to the formation of SA gels stabilized by extensive intermolecular hydrogen bonding. This protonation-induced network formation further promotes and reinforces the cross-linking of the hydrogel matrix. Subsequently, H^+^ ions react with CaCO_3_ within the hydrogel matrix, generating Ca^2+^ ions and CO_2_ gas in situ. The released Ca^2+^ ions further ionically cross-link the SA chains, while the concurrent evolution and outward diffusion of CO_2_ bubbles drive the formation of internal voids, ultimately giving rise to hollow tubular structures. Consequently, gas generation within the hydrogel plays a critical role in directing and stabilizing tube formation [9,33,34,37].

To further elucidate the internal microstructure of the hollow tubes formed by injecting the spinning precursor into gelation baths of varying pH, cross-sectional morphologies of the hydrogels were examined using scanning electron microscopy (SEM). As shown in Figure 1d,e, a well-defined central hollow cavity is clearly observed, confirming the successful formation of hollow tubular architectures. Although hollow hydrogel fabricated under different pH conditions exhibit distinct internal microstructures, all samples maintain a circular cross-sectional geometry. SEM analysis of the hollow hydrogels prepared at pH 0 (Figure 1d) reveals a porous wall morphology with pore sizes ranging from approximately 10 to 75 µm and an average pore size of 25 µm. The pore wall thickness is about 110 µm, while the inner hollow core diameter is approximately 225 µm. In contrast, the hollow hydrogels formed at pH 1 exhibit a comparatively dense wall structure with an average thickness of approximately 130 µm, as shown in Figure 1e. The observed differences in wall microstructure can be attributed to variations in CO_2_ generation and release behavior under different pH conditions. Specifically, the more vigorous gas evolution within the hydrogel matrix at pH 0 leads to extensive pore formation, resulting in highly porous SA tube walls. In addition to pH, the inner diameter (ID) and outer diameter (OD) of the hollow tubes are strongly governed by the molar ratio of calcium ions to carboxyl groups in the precursor system. The results reveal that the tube wall thickness decreases approximately linearly with increasing SA/Ca^2+^ ratio, as illustrated in Figure 1f. Increasing the CaCO_3_ content enhances the extent of CO_2_ generation during the acid–carbonate reaction, thereby promoting expansion of the inner cavity and resulting in a larger inner diameter. Simultaneously, the elevated release of Ca^2+^ ions increases the ionic cross-linking density of the SA network, leading to greater gel contraction and, consequently, a reduction in tube wall thickness.

As anticipated, the molar ratio of SA/Ca^2+^ strongly influences the mechanical strength of the hydrogels. As shown in Figure 1g, at low CaCO_3_ loadings (molar ratio 2:1 and 3:2), the pH 0 samples consistently exhibit higher stress values than their pH 1 counterparts. This behavior arises because the pH 0 environment contains approximately an order of magnitude higher H^+^ concentration, which accelerates CaCO_3_ dissolution and leads to a greater release of Ca^2+^ ions. The resulting higher Ca^2+^ availability promotes the formation of denser “egg-box” ionic cross-linking networks between Ca^2+^ and SA carboxyl groups [38], thereby enhancing the mechanical strength of the hydrogel. At a molar ratio of 1:1, the pH 1 samples display a pronounced increase in mechanical strength, reaching a peak stress of approximately 0.8 MPa and surpassing that of the pH 0 group. At this higher CaCO_3_ content, sufficient Ca^2+^ ions are available to achieve extensive cross-linking, while the relatively moderate H^+^ concentration in the pH 1 environment allows for a more controlled Ca^2+^ release. This balanced ion release promotes the formation of a uniform, densely packed cross-linked network, ultimately resulting in superior mechanical performance. In contrast, the excessively rapid release of Ca^2+^ in the pH 0 environment leads to localized over-crosslinking, resulting in the formation of brittle domains that compromise the mechanical integrity of the hydrogel. At a molar ratio of 4:5, a decline in stress is observed in both pH conditions, as the excessive availability of Ca^2+^ induces non-uniform over-crosslinking within the SA network, thereby deteriorating overall mechanical performance. Such an excessively dense network structure increases material brittleness, reflecting the well-known trade-off between cross-linking density and toughness in polysaccharide-based hydrogels, and ultimately leads to reduced mechanical strength. Overall, the mechanical performance of SA/CaCO_3_ hydrogel is synergistically governed by both the kinetics of Ca^2+^ release and the total availability of Ca^2+^ ions.

In addition, the effect of C_SA_ content on the mechanical performance was systematically investigated. As the C_SA_ concentration increased to 4 wt.%, the hydrogel strength initially rose to approximately 1.5 MPa and then decreased (Figure 1h). This behavior can be attributed to the excessive increase in solution viscosity at higher C_SA_ loadings (Figure S2), which compromises the homogeneity of the SA/CaCO_3_ precursor mixture and leads to non-uniform cross-linking density within the resulting hydrogel network. Given the tunable mechanical properties achieved via optimized preparation conditions, a comparative evaluation with other reported systems was conducted to assess the performance of the hollow hydrogel tubes. As shown in Table S1, the hollow hydrogel exhibited excellent mechanical performance, surpassing that of most existing biopolymer-based hollow fibers or tubes. This superior mechanical advantage endows the hollow hydrogel tubes with promising applications as structural biomaterials.

### 2.2. Enhanced Water Retention, Freeze Resistance, and Mechanical Properties of the SA Hollow Hydrogel

**Figure 2:**
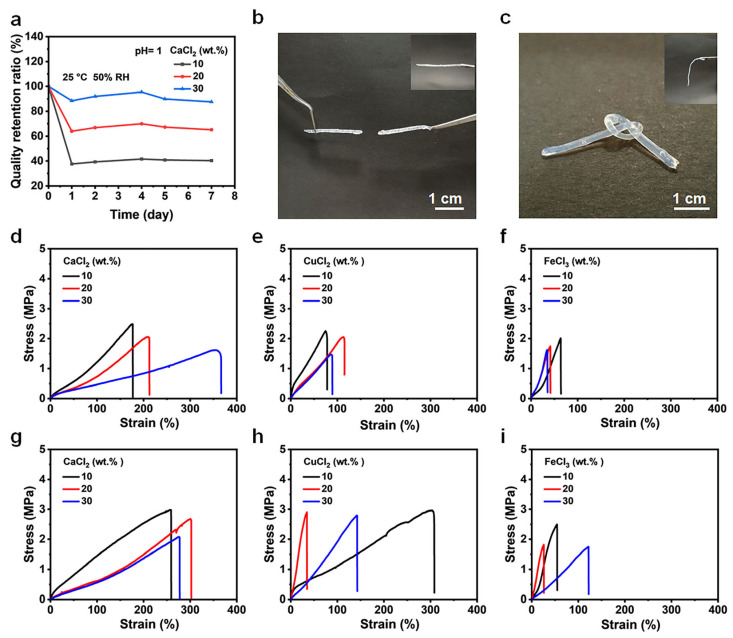
(**a**) Changes in quality retention ratio of hydrogels after secondary cross-linking with different concentrations of CaCl_2_ at 25 °C and 50% RH. Photographs of the hydrogel without (**b**) or with (**c**) secondary cross-linking after storage for 24 h at −20 °C. Stress–strain curves of hydrogels with different cross-linking ions (each at various concentrations) measured at room temperature: (**d**) CaCl_2_, (**e**) CuCl_2_, (**f**) FeCl_3_. Stress–strain curves of hydrogels frozen at −20 °C with different cross-linking ions (each at various concentrations) measured at room temperature: (**g**) CaCl_2_, (**h**) CuCl_2_, (**i**) FeCl_3_. (Hydrogel prepared at 4 wt.% SA, SA/Ca^2+^ = 1:1 and pH 1 before secondary crosslinking).

**Figure 3:**
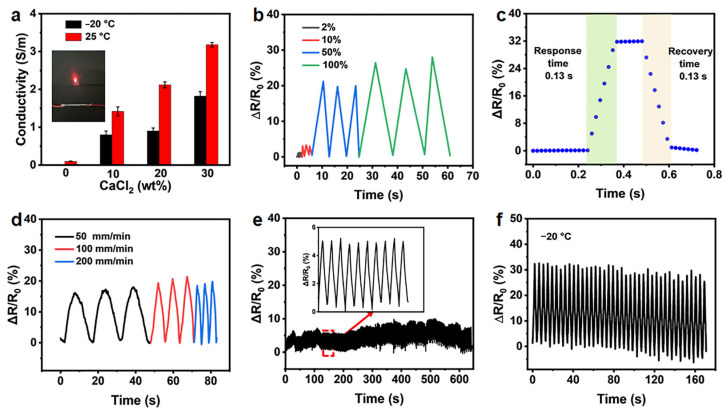
(**a**) The conductivity of hydrogels cross-linked with different concentration CaCl_2_ at different temperatures. (**b**) Time-dependent relative resistance changes of the sensors at small strain and lager strain. (**c**) The response and recovery time of the hydrogel strain sensor. (**d**) Relative resistance changes under a 50% stretch strain at different stretch rates. (**e**) Stability of relative resistance changes of the sensors for 300 cycles at 20% strain under the applied voltage of 0.1 V. (**f**) Relative resistance change stability of 30% CaCl_2_ secondary cross-linked hydrogel sensors after freezing treatment at −20 °C (0.1 V, 20% strain, 40 cycles).

Owing to residual, uncoordinated carboxylate groups, the as-fabricated hollow hydrogels can undergo secondary ionic cross-linking upon immersion in CaCl_2_ solutions of varying concentrations. This post-treatment markedly improves their water retention capability, antifreezing performance, and overall mechanical properties. Progressively increasing the CaCl_2_ concentration from 10% to 30% led to a steady improvement in the water retention capacity of the hydrogels, reaching a maximum value of approximately 87% after 7 days at 30% CaCl_2_ treatment, as shown in Figure 2a. The improved water retention performance can be attributed to the formation of calcium alginate ‘egg-box’ architectures, in which Ca^2+^ ions ionically bridge neighboring alginate chains, thereby restricting water diffusion, as well as to additional hydrogen bond interactions between Ca^2+^ ions and hydroxyl groups within the polymer matrix. To further clarify the origin of water retention, the leaching behavior of free CaCl_2_ was evaluated by electrical conductivity measurements. The initial electrical conductivity was determined to be 3.18 S/m (Figure 3a), and a conductivity decrease of approximately 41% was observed after immersion into water, verifying that free CaCl_2_ leached out from the hydrogel. Despite the leaching of free CaCl_2_, the hydrogel still maintained a low swelling ratio of about 8.3% (Figure S3) and excellent structural stability. These results clearly confirm that the outstanding water retention mainly originates from the stable egg-box cross-linked network and hydrogen-bonding interactions.

Furthermore, secondary cross-linking imparts pronounced antifreezing capability to the as-fabricated hydrogel. As illustrated in Figure 2b,c, hydrogels lacking secondary cross-linking undergo rapid brittle fracture upon knotting at −20 °C, whereas the secondarily cross-linked hydrogels maintain structural integrity, tolerate knot deformation without failure, and preserve remarkable flexibility under subzero conditions. In addition to markedly enhancing freezing resistance, Ca^2+^ incorporation significantly improves the mechanical robustness of the hydrogel. To substantiate this effect, secondarily cross-linked hollow hydrogels were subjected to tensile testing using a universal mechanical testing system both before and after freezing at −20 °C. At ambient temperature, hydrogels immersed in 10, 20, and 30 wt.% CaCl_2_ solutions exhibit comparable tensile stress–strain profiles, with the highest tensile strength (2.5 MPa) observed for the 10 wt.% CaCl_2_-treated sample. Notably, all secondarily cross-linked hydrogels demonstrate superior mechanical strength relative to the hydrogel without secondary cross-linking (Figure 2d). The improvement in mechanical performance induced by secondary cross-linking is predominantly attributed to strong coordination interactions between Ca^2+^ ions and the residual carboxylate groups along the SA chains. These coordination interactions generate additional ionic cross-linking nodes, resulting in a denser, more compact polymer network with enhanced chain entanglement and load-bearing capacity. Free Ca^2+^ ions from the 10 wt.% CaCl_2_ solutions diffuse uniformly throughout the hydrogel matrix and coordinate with previously uncross-linked carboxylate groups, forming additional stable ionic cross-linking points. This homogeneous increase in cross-linking density effectively reinforces the mechanical strength of the hydrogel while avoiding excessive over-crosslinking that could otherwise lead to brittleness.

In contrast, when the CaCl_2_ concentration exceeds 10 wt.% (i.e., 20 and 30 wt.%), non-uniform ionic cross-linking and localized over-densification of the polymer network are likely to occur. This structural heterogeneity progressively compromises mechanical performance relative to the optimal 10 wt.% condition, although the resulting strength remains higher than that of the hydrogel without secondary cross-linking. Figure 2g presents the tensile strength of hydrogels treated with varying CaCl_2_ concentrations, which was tested after freezing treatment at −20 °C, revealing a 20–30% increase in strength relative to measurements conducted at room temperature. This enhancement in mechanical performance is attributed to the formation of a low-temperature “slurry-like” microstructure, which improves load transfer and structural integrity [39]. In addition, Cu^2+^ and Fe^3+^ ions are capable of forming coordination cross-links with SA [40], thereby further reinforcing the mechanical performance of the hollow hydrogel while simultaneously conferring antifreezing functionality, as evidenced in Figure 2e,f,h,i and Figure S4. However, hydrogels cross-linked with Cu^2+^ or Fe^3+^ ions display reduced ductility compared with their Ca^2+^-cross-linked counterparts. From a practical standpoint, the salt type and concentration can be carefully tailored to balance stretchability and fracture toughness within the intended operating temperature range.

### 2.3. Sensing Properties of the SA Hollow Hydrogel

The hydrogels exhibit appreciable ionic conductivity of 0.1 S m^−1^ at 25 °C (Figure 3a). Utilizing this intrinsic ionic conduction, the SA hollow hydrogel shows strong potential as a flexible sensing material. Upon exposure to external mechanical stimuli, hydrogel deformation induces measurable variations in electrical resistance or conductivity, enabling reliable signal transduction. The relative change in electrical resistance was employed as the sensing signal to quantify strain variation, defined as ΔR/R_0_ = (R − R_0_)/R_0_, where ΔR represents the resistance change induced by deformation, R denotes the resistance under applied strain, and R_0_ corresponds to the initial resistance of the undeformed hydrogel.

Figure 3b illustrates the characteristic electrical response of the SA hydrogel sensor under small and large applied strains. The results reveal a monotonic increase in relative electrical resistance with increasing tensile strain, confirming that the fabricated hydrogels exhibit a clear, reproducible electromechanical response to mechanical deformation. The hydrogel sensor exhibits a rapid response time of approximately 0.13 s during both loading and unloading cycles (Figure 3c), which outperforms most currently reported SA-based hydrogels [32,41,42,43]. This short response time demonstrates the material’s ability to respond to external stimuli swiftly, enabling real-time monitoring of dynamic sensing signals. As depicted in Figure 3d, the SA hydrogel sensor delivers stable, reproducible signal amplitudes at identical strain levels across stretching rates of 50, 100, and 200 mm min^−1^, demonstrating rate-independent sensing behavior. Signal stability is a critical criterion for assessing the practical applicability of hydrogel-based sensors. As shown in Figure 3f, the hydrogel sensor maintains stable, repeatable electrical responses over 300 consecutive stretching–relaxation cycles at a tensile strain of 20%, demonstrating high strain-sensing sensitivity, excellent operational stability, and fatigue resistance. Moreover, the secondary cross-linked hydrogels retain effective ionic conductivity at temperatures well below the freezing point of water (Figure 3a). Coupled with their preserved stretchability and mechanical toughness under subzero conditions, the electromechanical loading−unloading curves of the secondary cross-linked hydrogel after freezing treatment at −20 °C, are stable over 40 cycles at a small strain of 20%. This capability broadens their applicability for sensing and functional operations in cold environments.

**Figure 4:**
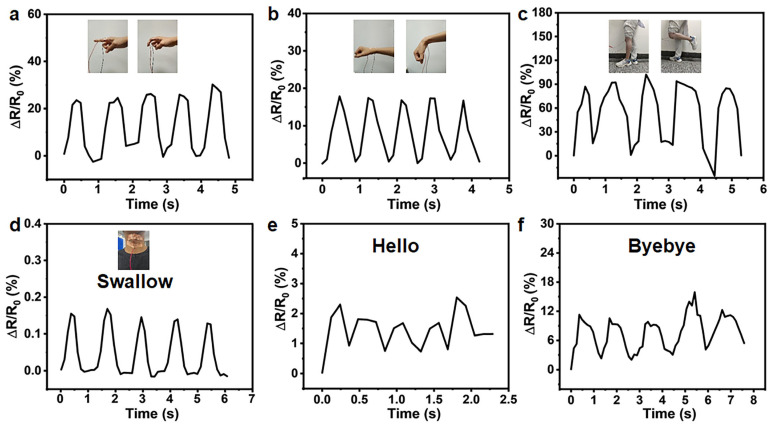
Relative resistance changes of sensors versus time for real-time monitoring of various human motions. (**a**–**c**) Bending and release of a finger, a wrist, and a knee, respectively. (**d**) Subtle muscle movements of the throat, such as swallowing. Subtle muscle movements of the throat as the person says the word (**e**) “hello” and (**f**) “bye-bye”.

Owing to its broad strain-sensing range, the fabricated SA hollow hydrogel can be directly integrated onto human skin for motion detection. As illustrated in Figure 4, progressive bending of body joints, including fingers, wrists, knees, and the neck, during stepwise grasping and movement induces distinct, incremental increases in relative electrical resistance, demonstrating the sensor’s ability to transduce diverse human motions into measurable electrical signals reliably. The relative resistance variations are fully synchronized with the repetitive motions of the fingers, wrists, knees, and neck, indicating reliable, real-time motion tracking. Furthermore, the SA hollow hydrogel can detect subtle physiological activities, such as swallowing and speech-related movements, as demonstrated in Figure 4d–f. Upon three consecutive repetitions of identical words by the same individual, the time-resolved relative resistance responses display highly consistent peak-valley patterns, indicating excellent signal repeatability. These results demonstrate that the SA hollow hydrogel possesses robust human motion sensing capabilities and holds strong promise for advanced wearable sensing applications.

### 2.4. SA Hollow Hydrogel with Complicated Shape

**Figure 5:**
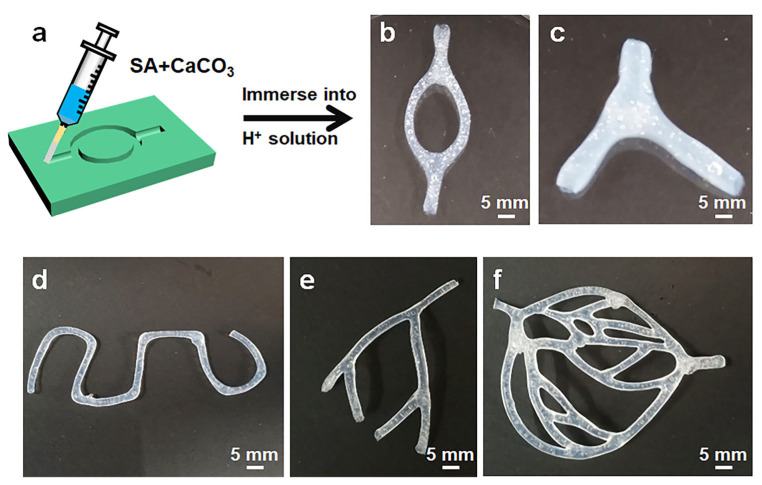
(**a**,**b**) Process of fabricating a connecting tubular structure. Examples of fabricated hollow hydrogel structures, including bifurcate (**c**), tortuous single-channel (**d**), quadrifurcate vasculature-like structure (**e**) and sophisticated circulatory system of blood vessels (**f**). (Hydrogel prepared at 4 wt.% SA, SA/Ca^2+^ = 1:1 and pH 1).

Beyond the facile fabrication of individual hollow hydrogels, the present strategy is highly versatile for constructing SA hollow hydrogels with complex 3D architectures. As illustrated in Figure 5a, a straightforward, scalable preparation process is used to generate these hollow hydrogel structures. A controlled volume of the spinning precursor was injected into the prefabricated mold and subsequently immersed in hydrochloric acid for several hours. During this process, a Ca-alginate physical hydrogel was formed within the mold via ionic cross-linking induced by Ca^2+^ ions. The resulting cross-linked hollow hydrogel can be readily removed from the mold while preserving the mold-defined geometry. From a fabrication standpoint, virtually any hydrogel architecture can be realized provided an appropriate mold is available, including interconnected tubular networks, bifurcating networks, tortuous single-channel networks, quadrifurcate vasculature-like structures and even sophisticated circulatory system of blood vessels, as demonstrated in Figure 5b–f. These findings clearly demonstrate the versatility of the proposed strategy for fabricating diverse, complex-shaped, 3D freestanding hydrogel architectures.

### 2.5. Biocompatibility of the SA Hollow Hydrogel

**Figure 6:**
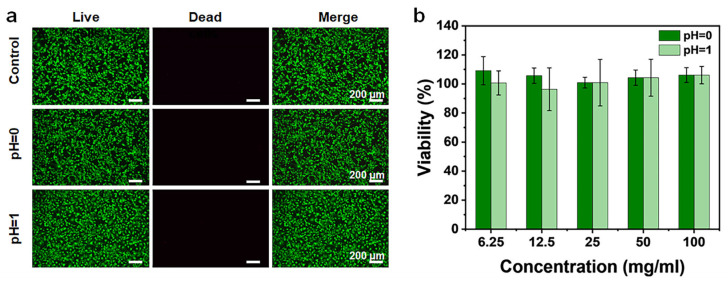
(**a**) Live/dead cell-stained fluorescent images of hydrogels prepared in coagulation baths with different pH values. (**b**) In vitro cytocompatibility evaluated with various dosages of hydrogel extracts.

Alginate hydrogels are among the most widely used biomaterials for biomedical applications due to their high permeability, excellent biocompatibility and biodegradability, low cytotoxicity, and capability for in situ ionic cross-linking [44,45]. Accordingly, the in vitro cytocompatibility of the SA hollow hydrogel was systematically assessed using a 3-(4,5-dimethylthiazol-2-yl)-2,5-diphenyltetrazolium bromide (MTT) assay. Fluorescence microscopy images revealed that after 24 h of incubation, cells maintained high viability, characterized by a uniform distribution of green-fluorescent live cells and only occasional red-fluorescent dead cells (Figure 6a). Consistently, MTT assay results showed that cell viability remained high (≥95%) across all tested extracts at different concentrations (Figure 6b), indicating negligible cytotoxicity of the SA hollow hydrogel. These findings confirm that the hydrogel tubes exhibit excellent biocompatibility and minimal cytotoxicity. In addition to cytocompatibility, the physiological stability of the hollow hydrogel tubes was further evaluated under simulated biological conditions. The swelling behavior, which is fundamental for tissue engineering performance, showed that water uptake increased progressively during the first 6 h and then reached equilibrium (Figure S5a,b). Specifically, after 6 h of immersion in phosphate-buffered solution (PBS), the hydrogels prepared at pH 0 and pH 1 exhibited swelling ratios of approximately 480% and 670%, respectively. More importantly, after immersion in PBS (pH 7.4) at 37 °C for up to one week, the hollow hydrogels retained their tubular structure and structural integrity with no obvious dissolution, collapse, or degradation, while their diameters increased to about 2.0-fold and 2.3-fold of the original values (Figure S6a,b). These results demonstrate that the hollow hydrogels possess sufficient structural stability for short-to-medium term applications under physiological conditions, despite undergoing significant initial swelling. Owing to their tunable physicochemical properties and well-defined hollow architecture, the hydrogel tubes developed in this study show strong potential for use as artificial blood vessels, emphasizing the critical role of hollow hydrogel systems in the engineering of tubular tissues.

## 3. Conclusions

In summary, a facile wet-spinning approach was developed to fabricate SA hollow hydrogels without reliance on complex processing steps. The resulting hollow hydrogels exhibit precisely controllable dimensions and a combination of outstanding properties, including tunable morphology and enhanced mechanical performance. These key properties can be readily tuned by modulating the pH of the coagulation bath and the concentrations of SA and CaCO_3_. Notably, the hollow hydrogel platform is highly versatile and can be easily functionalized to impart diverse functionalities. Secondary cross-linking endows the fabricated hollow hydrogels with enhanced water retention and antifreezing capabilities. At the same time, their excellent ionic conductivity enables reliable detection of both large-amplitude limb motions and subtle muscle activities in the human body. These attributes position the hydrogels as promising platforms for applications in skin-inspired electronics, human–machine interfaces, and artificial prosthetic systems. Moreover, a wide variety of 3D branched hollow architectures can be readily fabricated by simply modifying the mold design. In addition, the SA hollow hydrogels demonstrate excellent biocompatibility and minimal cytotoxicity, further highlighting their suitability for bioengineering applications. Overall, this work presents a versatile and scalable strategy for the rational design and fabrication of multifunctional hollow hydrogels, offering new opportunities for their widespread implementation in flexible electronics and advanced biomedical engineering.

## 4. Materials and Methods

### 4.1. Materials

Sodium alginate (SA powder, Mw = 1,171,546 Da; 60 mesh; dynamic viscosity of 450–550 mPa·s (1 wt.% aqueous solution at 25 °C) was provided by Qingdao Hyzlin Biology Development Co., Ltd. (Qingdao, China). Calcium carbonate (CaCO_3_) and hydrochloric acid (HCl) were purchased from a commercial supplier (Sinopharm Chemical Reagent Co., Ltd., Shanghai, China).

### 4.2. Preparation of Spinning Mixture

SA powder was added to deionized water stirring for 4 h at room temperature and then stirred at 50 °C for 0.5 h. After SA dissolved completely, CaCO_3_ was added under continuous stirring. The mixture was stirred 1 h to obtain a homogeneous spinning mixture. The detailed compositions of the mixtures are listed in the Table 1.

### 4.3. Preparation of SA Hollow Hydrogel

Hollow hydrogels were prepared by extruding the spinning mixture into solutions of hydrochloric acid at a pH of 0 (theoretical, 1 mol/L HCl) or 1 (theoretical, 0.1 mol/L HCl). After extrusion, the samples were immersed in the acid solution, resulting in the formation of SA hollow hydrogels. The secondary cross-linked hydrogels were prepared by immersing the above hydrogel (prepared at 4 wt.% SA, SA/Ca^2+^ = 1:1 and pH 1) into the 10, 20, 30 wt.% CaCl_2_ solutions for 24 h, respectively.

### 4.4. Characterizations

#### 4.4.1. Morphological Characterization

The scanning electron microscopy (SEM) images of hydrogels were acquired by Zeiss Ultra 55 apparatus (Carl Zeiss AG, Oberkochen, Baden-Württemberg, Germany) using the freeze-dried sample at an acceleration voltage of 3 kV. The cross-section of the hydrogel was sprayed with a layer of gold before the test.

#### 4.4.2. Mechanical Strength Test

Tensile tests were performed on a universal mechanical tester (WDW-5T, Jinan, China) with a 250 N load cell at a strain rate of 5 mm/min at room temperature.

#### 4.4.3. Rheological Measurement

Rheological measurement of the SA solution viscosity was implemented on a rotational rheometer (MCR301, Anton Paar Instrument, Anton-Paar-Straße 20, 8054 Graz, Austria), utilizing a 25 mm-diameter cone-and-plate accessory with a 2° cone angle and a truncated gap of 0.05 mm. The shear rate was varied within the range of 0.01–1000 s^−1^ during the test, and the zero-shear viscosity was computed automatically by the software bundled with the instrument.

#### 4.4.4. Water Retention Capacity Characterization

The water retention tests were conducted at a constant temperature of 25 °C and a relative humidity (RH) of 50%, maintained using a climate chamber. The weight retention ratio (W_Ri_) is calculated using the following formula W_Ri_ = W_i_/W_0_. Herein, W_i_ and W_0_ correspond to the hydrogel weight at the i-th day and the initial weight (day 0), respectively.

#### 4.4.5. Water Uptake Test

The water uptake behavior of the hydrogels was evaluated by gravimetric analysis.

For the secondarily cross-linked hydrogels, the samples were immersed in deionized water at 25 °C. At predetermined time intervals, the samples were retrieved, gently blotted with filter paper to remove excess surface water, and immediately weighed using an analytical balance. The process was repeated until the sample weight reached a constant value, indicating equilibrium.

For the SA hollow hydrogels, the procedure was similar, except that the immersion medium was PBS (pH 7.4) and the temperature was maintained at 37 °C using a thermostatic water bath.

The water uptake ratio at each time point was calculated using the following equation: Water uptake (%) = (W_t_ − W_0_/W_0_) × 100%, where W_0_ is the initial weight of the hydrogel, and W_t_ is the weight at time. All measurements were performed in triplicate.

#### 4.4.6. Conductivity Measurement

The conductivity of hydrogels was measured by Keithley 2450 digital meter (Keithley Instruments, Inc., Cleveland, OH, USA). The conductivity (σ) was calculated using the following equation, σ = L/RS, in which R is the resistance, and L and S are the materials’ length and effective annular cross-sectional area, respectively.

#### 4.4.7. Characterization of SA Hollow Hydrogel Strain Sensors

The SA hydrogel sample was clamped with two copper sheets and fixed on a universal mechanical tester (WDW-5T, China) for stretching, while the electrical signals of the strain sensors were recorded at the same time by a Keithley 2450. For monitoring the human movement, the strain sensors were attached onto various joints of the human body, including fingers, wrist, knees, and throat. The relative change of the resistance was calculated as follows: ∆R/R_0_ = (R − R_0_)/R_0_, where R_0_ and R are the resistance without and with applied strain, respectively.

#### 4.4.8. Cytocompatibility Test

The hydrogel extracts were obtained by soaking sterilized hydrogel in Dulbecco’s modified Eagle medium (DMEM) culture medium at 37 °C for 24 h, followed by filtration through a 0.22 μm filter. NIH-3T3 mouse fibroblast cells were seeded in a 96-well plate at 104 cells/well in 100 μL of culture medium, and maintained at 37 °C in a 5% CO_2_ environment for 12 h. Then, the medium was replaced with hydrogel extracts (6.25, 12.5, 25, 50, 100 mg/mL), using the culture medium as a control. After 24 h of incubation, cells were treated with MTT (10 μL/well, 5 mg/mL) and incubated for an additional 4 h. Subsequently, the MTT solution was removed, and dimethyl sulfoxide (150 μL/well) was added to each well. The plates were then shaken gently for 20 min. The absorbance at 490 nm was recorded with a microplate reader for the calculation of cell viability (CV). CV was calculated using the following formula: CV (%) = Atest/Acontrol × 100%, Atest represents the absorbance of the hydrogel sample group, and Acontrol represented the control group. Additionally, cells were stained with calcein-AM/PI and imaged using an inverted fluorescence microscope.

## Figures and Tables

**Table 1:** The compositions of the spinning mixtures.

SA Mass Concentration	Molar Ratio SA:CaCO_3_	SA/g	CaCO_3_/g	H_2_O/g
2 wt.%	2:1	2.00	0.51	97.49
2 wt.%	3:2	2.00	0.67	97.33
2 wt.%	1:1	2.00	1.01	96.99
2 wt.%	4:5	2.00	1.26	96.74
3 wt.%	1:1	3.00	1.52	95.48
4 wt.%	1:1	4.00	2.02	93.98
5 wt.%	1:1	5.00	2.53	92.47

## Data Availability

The original contributions presented in the study are included in the article/Supplementary Material. Further inquiries can be directed to the corresponding author.

## Supplementary Materials

The following supporting information can be downloaded at: https://www.mdpi.com/article/10.3390/gels12030224/s1, Figure S1: State of the SA/CaCO_3_ extrudate in coagulation baths with different pH; Figure S2: Viscosity at different SA Concentrations; Figure S3: Swelling behavior of 30 wt.% CaCl_2_ cross-linked SA hollow hydrogels; Figure S4: Photographs of the hydrogel with Cu^2+^ (a) or Fe^3+^ (b) secondary cross-linking after storage for 24 h at −20 °C; Figure S5: Swelling ability of SA hollow hydrogels in PBS at 37 °C for (a) 9 h and (b) 7 d; Figure S6: Optical images of SA hollow hydrogel before (a) and after (b) immersion in PBS (pH 7.4) at 37 °C for one week; Table S1: Comparison of mechanical properties with other biopolymer-based hollow fibers or tubes.

## References

[1] Nandakumar D.K., Ravi S.K., Zhang Y.X., Guo N., Zhang C., Tan S.C. (2018). A super hygroscopic hydrogel for harnessing ambient humidity for energy conservation and harvesting. Energy Environ. Sci..

[2] Liu X., Tang T.-C., Tham E., Yuk H., Lin S., Lu T.K., Zhao X. (2017). Stretchable living materials and devices with hydrogel–elastomer hybrids hosting programmed cells. Proc. Natl. Acad. Sci. USA.

[3] Ullah S., Khattak S., Rahim F., Zheng Q., Ma J., Zahra Q.U.A., Xu H.-T., Shen J. (2026). ECM-inspired mechanically robust hydrogels for diabetic wound regeneration. Coord. Chem. Rev..

[4] Cui H., Pan N., Fan W., Liu C., Li Y., Xia Y., Sui K. (2019). Ultrafast Fabrication of Gradient Nanoporous All-Polysaccharide Films as Strong, Superfast, and Multiresponsive Actuators. Adv. Funct. Mater..

[5] Nasseri R., Bouzari N., Huang J., Golzar H., Jankhani S., Tang X., Mekonnen T.H., Aghakhani A., Shahsavan H. (2023). Programmable nanocomposites of cellulose nanocrystals and zwitterionic hydrogels for soft robotics. Nat. Commun..

[6] Chen J., Liu F., Abdiryim T., Liu X. (2024). An overview of conductive composite hydrogels for flexible electronic devices. Adv. Compos. Hybrid Mater..

[7] Maiti S., Maji B., Yadav H. (2024). Progress on green crosslinking of polysaccharide hydrogels for drug delivery and tissue engineering applications. Carbohydr. Polym..

[8] Chen Q., Liang S., Chen T., Zhang L. (2025). Engineering Hollow Hydrogel Architectures toward Cutting-Edge Applications. Adv. Healthc. Mater..

[9] Liang S., Tu Y., Chen Q., Jia W., Wang W., Zhang L. (2019). Microscopic hollow hydrogel springs, necklaces and ladders: A tubular robot as a potential vascular scavenger. Mater. Horiz..

[10] Liang S., Al-Handawi M.B., Chen T., Naumov P., Zhang L. (2025). Hollow Hydrogels for Excellent Aerial Water Collection and Autonomous Release. Angew. Chem. Int. Ed. Engl..

[11] Deng P., He Z., Shen Y., Mohammad N.M., Xu W., Han B., Li T. (2024). Conductive hollow hydrogel fibers toward high-sensitivity bio-textiles. Cell Rep. Phys. Sci..

[12] Pi Q., Maharjan S., Yan X., Liu X., Singh B., van Genderen A.M., Robledo-Padilla F., Parra-Saldivar R., Hu N., Jia W. (2018). Digitally Tunable Microfluidic Bioprinting of Multilayered Cannular Tissues. Adv. Mater..

[13] Cheng Y., Zheng F., Lu J., Shang L., Xie Z., Zhao Y., Chen Y., Gu Z. (2014). Bioinspired Multicompartmental Microfibers from Microfluidics. Adv. Mater..

[14] Wang D., Maharjan S., Kuang X., Wang Z., Mille L.S., Tao M., Yu P., Cao X., Lian L., Lv L. (2022). Microfluidic bioprinting of tough hydrogel-based vascular conduits for functional blood vessels. Sci. Adv..

[15] Xie R., Liang Z., Ai Y., Zheng W., Xiong J., Xu P., Liu Y., Ding M., Gao J., Wang J. (2021). Composable microfluidic spinning platforms for facile production of biomimetic perfusable hydrogel microtubes. Nat. Protoc..

[16] Rojek K.O., Ćwiklińska M., Kuczak J., Guzowski J. (2022). Microfluidic formulation of topological hydrogels for microtissue engineering. Chem. Rev..

[17] Xu P., Xie R., Liu Y., Luo G., Ding M., Liang Q. (2017). Bioinspired Microfibers with Embedded Perfusable Helical Channels. Adv. Mater..

[18] Wang Y., Kankala R.K., Zhu K., Wang S.B., Zhang Y.S., Chen A.Z. (2019). Coaxial Extrusion of Tubular Tissue Constructs Using a Gelatin/GelMA Blend Bioink. ACS Biomater. Sci. Eng..

[19] Lyu Y., Ji Z.Y., Liu D., Xu X., Guo R., Shic X., Wang X. (2025). Spider-silk inspired ultrafast alkali-induced molecular aggregation for 3D printing arbitrary tubular hydrogels. Mater. Horiz..

[20] Janarthanan G., Lee S., Noh I. (2021). 3D Printing of Bioinspired Alginate-Albumin Based Instant Gel Ink with Electroconductivity and Its Expansion to Direct Four-Axis Printing of Hollow Porous Tubular Constructs without Supporting Materials. Adv. Funct. Mater..

[21] Liang Q., Gao F., Zeng Z., Yang J., Wu M., Gao C., Cheng D., Pan H., Liu W., Ruan C. (2020). Coaxial Scale-Up Printing of Diameter-Tunable Biohybrid Hydrogel Microtubes with High Strength, Perfusability, and Endothelialization. Adv. Funct. Mater..

[22] Ying G., Jiang N., Parra-Cantu C., Tang G., Zhang J., Wang H., Chen S., Huang N., Xie J., Zhang Y. (2020). Bioprinted Injectable Hierarchically Porous Gelatin Methacryloyl Hydrogel Constructs with Shape-Memory Properties. Adv. Funct. Mater..

[23] Silva J.M., Duarte A.R.C., Custódio C.A., Sher P., Neto A.I., Pinho A.C.M., Fonseca J., Reis R.L., Mano J.F. (2014). Mano Nanostructured hollow tubes based on chitosan and alginate multilayers. Adv. Healthc. Mater..

[24] Zhang X., Zhao J., Xia T., Li Q., Ao C., Wang Q., Zhang W., Lu C., Deng Y. (2020). Hollow polypyrrole/cellulose hydrogels for high-performance flexible supercapacitors. Energy Storage Mater..

[25] Imani K.B.C., Kim D., Kim D., Yoon J. (2018). Temperature-Controllable Hydrogels in Double-Walled Microtube Structure Prepared by Using a Triple Channel Microfluidic System. Langmuir.

[26] Sun J., Schmidt B.V.K.J., Wang X., Shalom M. (2017). Self-standing carbon nitride-based hydrogels with high photocatalytic activity. ACS Appl. Mater. Interfaces.

[27] Lin H., Ma S., Yu B., Cai M., Zheng Z., Zhou F., Liu W. (2019). Fabrication of asymmetric tubular hydrogels through polymerization-assisted welding for thermal flow actuated artificial muscles. Chem. Mater..

[28] Mori N., Morimoto Y., Takeuchi S. (2017). Skin integrated with perfusable vascular channels on a chip. Biomaterials.

[29] Luo C., Guo A., Zhao Y., Sun X. (2022). A high strength, low friction, and biocompatible hydrogel from PVA, chitosan and sodium alginate for articular cartilage. Carbohydr. Polym..

[30] Huang X., Yu W., Gu W., Liang S., Zhou L., Zhang L. (2025). Mimicking natural biomineralization enabling biodegradable and highly lipophobic alginate hydrogels. Carbohydr. Polym..

[31] Tong R., Ma Z., Yao R., Gu P., Li T., Liu L., Guo F., Zeng M., Xu J. (2023). Stretchable and transparent alginate ionic gel film for multifunctional sensors and devices. Int. J. Biol. Macromol..

[32] Zhang X., Sheng N., Wang L., Tan Y., Liu C., Xia Y., Nie Z., Sui K. (2019). Supramolecular nanofibrillar hydrogels as highly stretchable, elastic and sensitive ionic sensors. Mater. Horiz..

[33] Chen Q., Tan H., Tu Y., Zhang L. (2019). Experimental insight into the evolutionary mechanism of solid-to-hollow hydrogel. Chem. Commun..

[34] Chen Q., Liang S., Song X., Naumov P., Zhang L. (2018). Hollow hydrogel networks for temperature-controlled water fluidics. Chem. Commun..

[35] Haug A., Myklestad S., Larsen B., Smidsrød O. (1967). Correlation between Chemical Structure and Physical Properties of Alginates. Acta Chem. Scand..

[36] Chan L.W., Lee H.Y., Heng P.W.S. (2006). Mechanisms of external and internal gelation and their impact on the functions of alginate as a coat and delivery system. Carbohydr. Polym..

[37] Tu Y., Chen Q., Liang S., Zhao Q., Zhou X., Huang W., Huang X., Zhang L. (2019). Antifreezing Heat-Resistant Hollow Hydrogel Tubes. ACS Appl. Mater. Interfaces.

[38] Cao L., Lu W., Mata A., Nishinari K., Fang Y. (2020). Egg-box model-based gelation of alginate and pectin: A review. Carbohydr. Polym..

[39] Morelle X.P., Illeperuma W.R., Tian K., Bai R., Suo Z., Vlassak J.J. (2018). Highly Stretchable and Tough Hydrogels below Water Freezing Temperature. Adv. Mater..

[40] Silva J., Vanat P., Marques-da-Silva D., Rodrigues J.R., Lagoa R. (2020). Metal alginates for polyphenol delivery systems: Studies on crosslinking ions and easy-to-use patches for release of protective flavonoids in skin. Bioact. Mater..

[41] Zhang X., Zhang J., Liao W., Zhang D., Dai Y., Wu C., Wen J., Zeng W. (2023). Stretchable conductive hydrogels integrated with microelectronic devices for strain sensing. J. Mater. Chem. C.

[42] Qiao H., Qi P., Zhang X., Wang L., Tan Y., Luan Z., Xia Y., Li Y., Sui K. (2019). Multiple Weak H-Bonds Lead to Highly Sensitive, Stretchable, Self-Adhesive, and Self-Healing Ionic Sensors. ACS Appl. Mater. Interfaces.

[43] Wang Q., Yu J., Lu X., Cao S., Chen L., Pan X., Ni Y., Ma X. (2021). 3D hollow-structured hydrogels with editable macrostructure, function, and mechanical properties induced by segmented adjustments. RSC Adv..

[44] Bäumchen A., Balsters J.M., Nenninger B.-S., Diebels S., Zimmermann H., Roland M., Gepp M.M. (2025). Towards a Comprehensive Framework for Made-to-Measure Alginate Scaffolds for Tissue Engineering Using Numerical Simulation. Gels.

[45] Park H., Lee H.J., An H., Lee K.Y. (2017). Alginate hydrogels modified with low molecular weight hyaluronate for cartilage regeneration. Carbohydr. Polym..

