# Supplementary material for: Template-Free Wet-Spinning of Multifunctional Sodium Alginate Hollow Hydrogels
Source: Gels. 2026 Mar 10;12(3):224. doi: 10.3390/gels12030224 (PMC13026051; doi:10.3390/gels12030224)
Supplement: Supplementary file 1 [file gels-12-00224-s001.zip › gels-4166665-supplementary.pdf]

Template-Free Wet-Spinning of Multifunctional Sodium Alginate Hollow Hydrogels

Na Pan [1,*], Haoran Sun [2] and Yanhu Zhan [1]

[1] School of Materials Science and Engineering, Liaocheng University, Liaocheng 252000, China

[2] School of Chemistry and Chemical Engineering, Liaocheng University, Liaocheng 252000, China

[*] Corresponding authors.
E-mail address: N. Pan (napan@lcu.edu.cn)

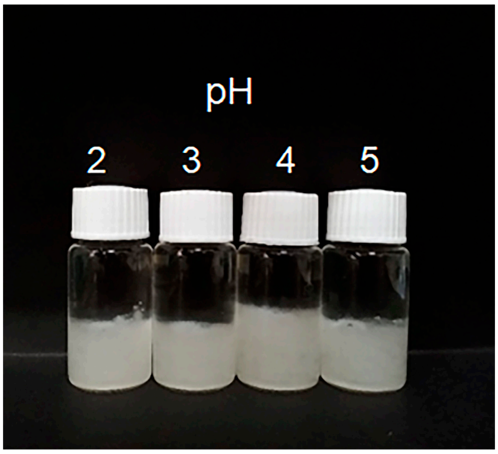

Figure S1. State of the SA/$CaCO_3$ extrudate in coagulation baths with different pH.

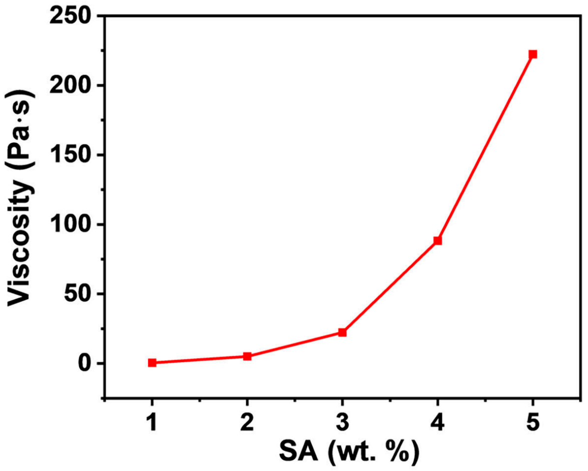

Figure S2. Viscosity at different SA Concentrations.

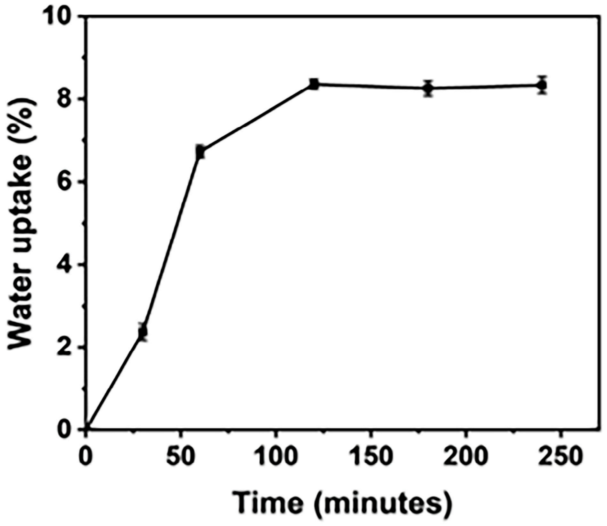

Figure S3. Swelling behavior of 30 wt.% $CaCl_2$ cross-linked SA hollow hydrogels.

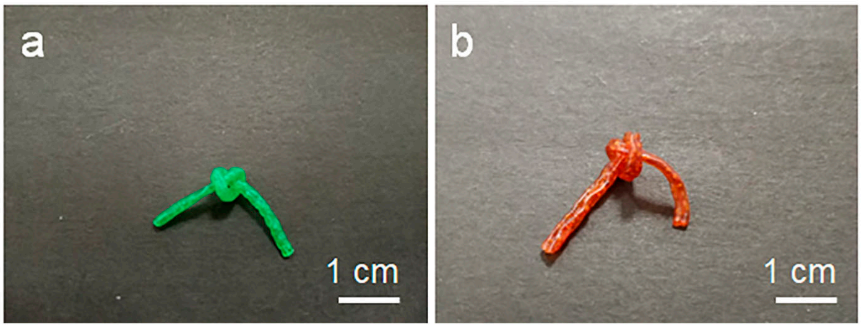

Figure S4. Photographs of the hydrogel with Cu^{2+} **(a)** or Fe^{3+} **(b)** secondary cross-linking after storage for 24 h at -20 °C.

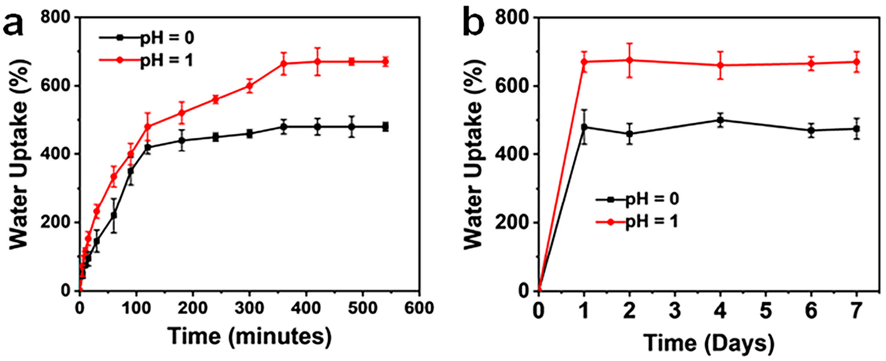

Figure S5. Swelling ability of SA hollow hydrogels in PBS at 37 °C for **(a)** 9 h and **(b)** 7 d.

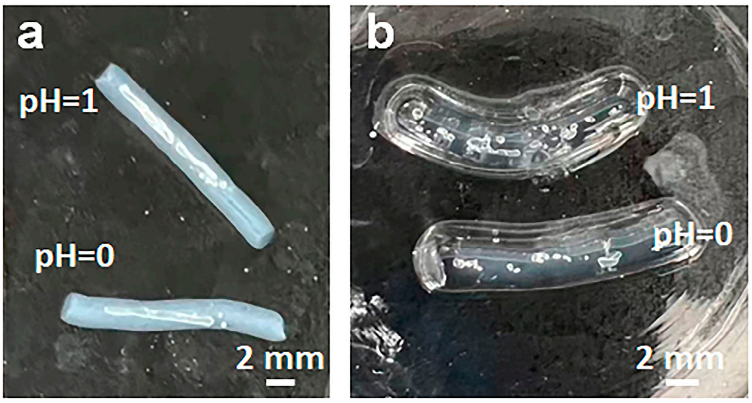

Figure S6. Optical images of SA hollow hydrogel before **(a)** and after **(b)** immersion in PBS (pH 7.4) at 37 °C for one week.

Table S1. Comparison of mechanical properties with other biopolymer-based hollow fibers or tubes.

Composition	Stress (MPa)	Ref.
SA	1.5	This work
PVA–G-FT	0.55	RSC Adv., 2021, 11, 26876–26882
ALG/CHIT	0.052	Adv. Healthcare Mater, 2014, 3, 433-440
SA	0.5	ACS Appl. Mater. Interfaces 2024, 16, 23, 29600–29609
SA	1.1	Chem. Commun., 2018, 54, 10304--10307
SA	3.4	ACS Appl. Mater. Interfaces 2019, 11, 18746−18754
SA	0.25	Mater. Horiz., 2019, 6, 2135--2142
AP-PVA	1.15	ACS Nano 2024, 18, 37, 25765–25777
